# Supplementary material for: Differentiation shifts from a reversible to an irreversible heterochromatin state at the DM1 locus
Source: Nat Commun. 2024 Apr 16;15:3270. doi: 10.1038/s41467-024-47217-4 (PMC11021500; doi:10.1038/s41467-024-47217-4)
Supplement: Supplementary file 1 — Supplementary Information [file 41467_2024_47217_MOESM1_ESM.pdf]

Figure S1 - Deletion of a large CTG repeat in DM1 hESC line.

a Schematic overview illustrating the CRISPR/Cas9 target sites (dashed vertical lines) relative to the CTG repeat (blue bars), and the PCR primers utilized for the initial screen (resulting in a 262 bp product from the intact 5 CTG allele or a 189 bp product if targeting was successful), and for validating targeting efficiency (the 567 bp product was expected if not efficient). Black and green asterisks mark the predicted breakpoints in hESCs (7gRNA and 44gRNAs) and myoblasts (27), respectively.

b Gene Scan analysis of PCR products from DM1-affected (SZ-DM14), gene edited clones (Δ/Δ CL9 and CL29), and wild type (SZ-RB26) hESCs enabled the validation of successfully targeted alleles, resulting in a 189 bp fragment due to the loss of a 72 bp product from the normal (represented by a 262 bp product) or expanded (undetected by PCR) alleles.

c PCR amplification of the cut region shows an absence of PCR products (567 bp) indicating homozygosity (clones CL9 and CL9), as opposed to heterozygosity (CL7 and CL29), for the deletion of the CTG repeat tract in genetically modified hESC clones and unmanipulated cells (SZ-DM14).

d DNA sequencing for validation of the CTG repeat excision; DNA sequencing of the CRISPRed targeted region in SZ-DM14 (CTG2000), before and after gene editing (CL9 and CL29), as well as in control hESCs (SZ-RB26, sequencing of complement strand, bottom panel). Black arrowhead indicate the site where the double strand breaks were fused.

Figure S1

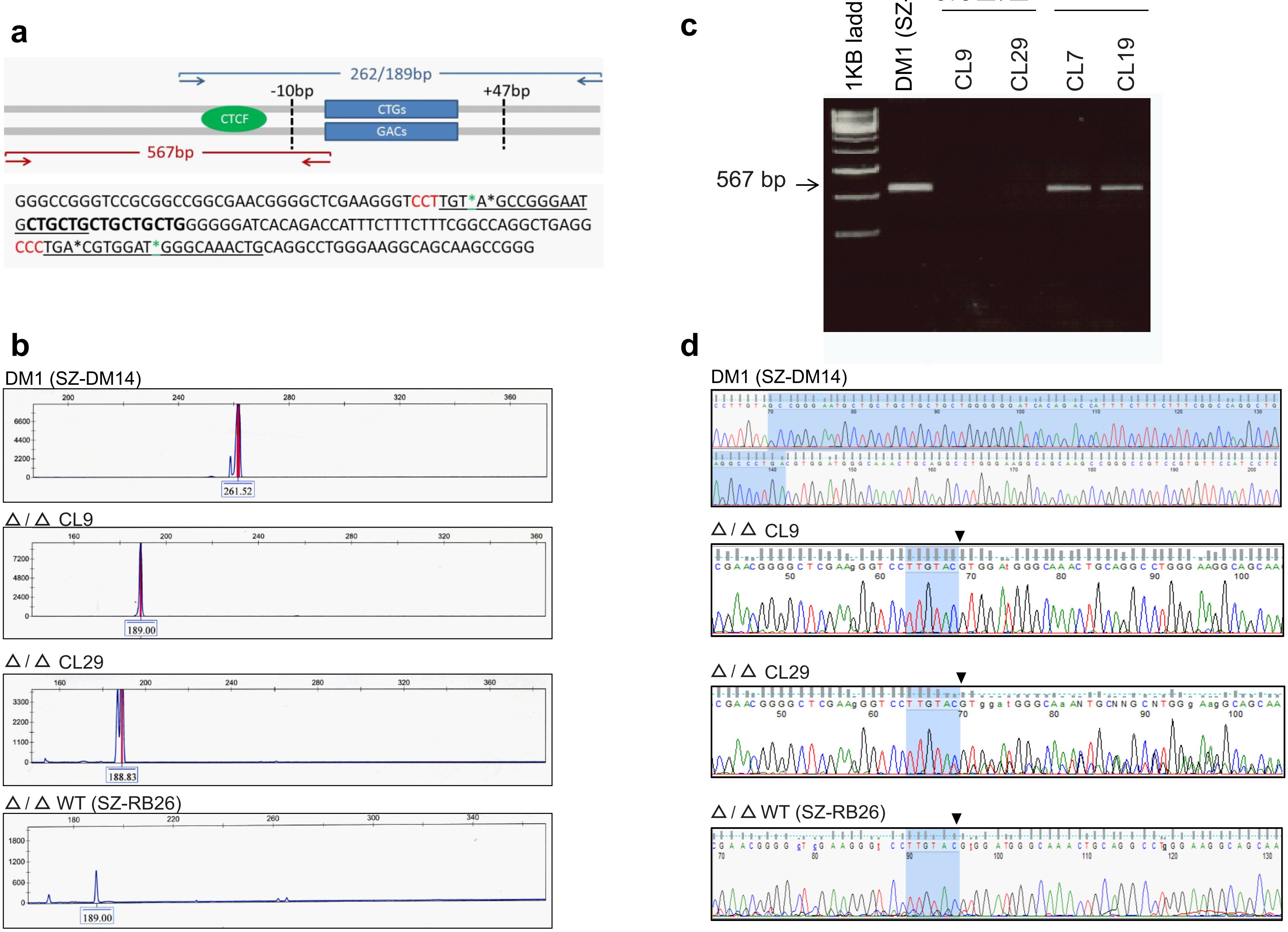
a
262/189bp
-10bp
+47bp
CTCF
CTGs
GACs
567bp
GGGCCGGGTCCGCGGCCGGCGAACGGGGCTCGAAGGGTCCTTGT*A*GCCGGGAAT
GCTGCTGCTGCTGCTGGGGGGATCACAGACCATTTCTTTCTTTCGGCCAGGCTGAGG
CCCTGA*CGTGGAT*GGGCAAACTGCAGGCCTGGGAAGGCAGCAAGCCGGG
b
DM1 (SZ-DM14)
261.52
Δ / Δ CL9
189.00
Δ / Δ CL29
188.83
Δ / Δ WT (SZ-RB26)
189.00
c
1KB ladder
DM1 (SZ-DM14)
CTG Δ / Δ
CTG + / Δ
CL9
CL29
CL7
CL19
567 bp
d
DM1 (SZ-DM14)
Δ / Δ CL9
Δ / Δ CL29
Δ / Δ WT (SZ-RB26)

Figure S2 - Validation of CTG repeat deletion and analysis of potential off-target sites.

a Validation of CTG excision by enzyme restriction length analysis detected by Southern blot in untransfected SZ-DM14 (2000 CTGs) cells and five representative clones after transfection, including CL9 and CL29 (Δ/Δ). Restriction analysis using this assay served to distinguish between normal alleles (~1.8 kb fragment with 5 CTGs), the original expanded alleles (~8.6 kb fragment with ~2000 CTGs), completely CTG-deleted alleles (~1.7 kb) and fragments between 1.8 and ~8.6 kb (representing mutant alleles that underwent partial deletion by the CRISPR/Cas9 system).

b Potential off-target sites for 7gRNA or 44gRNA (Table S2) were assessed by DNA sequencing to rule out the possibility of off-target effects. For each Δ/Δ clone, genomic DNA was isolated and analyzed for potential off-targets by Sanger sequencing of the PCR amplified products. For each off-target site, we present the sequence before (SZ-DM14) and after gene targeting (CL9 and CL29), aligned with a reference sequence, as it appears in the UCSC Genome Browser (GRCh38/hg38) Assembly. Note that no change in the DNA sequence was observed in any of the potential off-target sites.

Figure S2

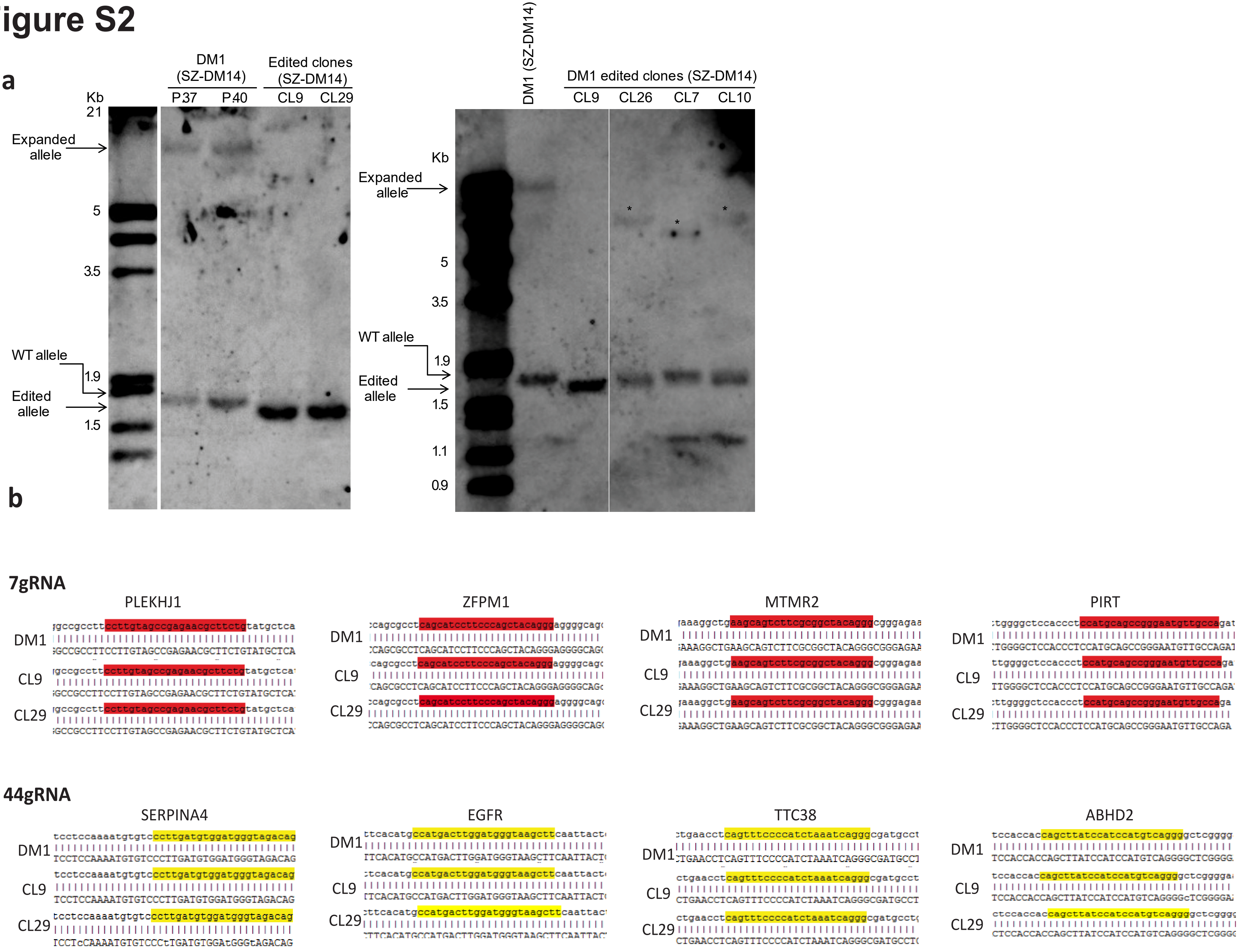
a
Kb
DM1 (SZ-DM14)
P37
P40
Edited clones (SZ-DM14)
CL9
CL29
21
Expanded allele
5
3.5
WT allele
1.9
Edited allele
1.5
DM1 (SZ-DM14)
DM1 edited clones (SZ-DM14)
CL9
CL26
CL7
CL10
Kb
Expanded allele
5
3.5
WT allele
1.9
Edited allele
1.5
1.1
0.9
b
7gRNA
PLEKHJ1
ZFPM1
MTMR2
PIRT
DM1
CL9
CL29
44gRNA
SERPINA4
EGFR
TTC38
ABHD2
DM1
CL9
CL29

Figure S3 - Validating hypomethylation, CTCF binding and local gene transcription in DM1 hESCs following gene editing.

a Heatmap shows methylation levels post-bisulfite deep-sequencing at the DMR (15 CpG sites) in DM1 hESCs (SZ-DM14) and gene-edited clones (CL9 and CL29).

b Colony DNA bisulfite sequencing at the CTCF binding site upstream of the repeats (17 CpGs) in wild-type hESCs with 5/11 CTG alleles (WT), unmanipulated hESCs (SZ-DM14), and gene-edited CTG-deficient (Δ/Δ) clones (CL9 and CL29). Filled circles: methylated CpGs; empty circles: unmethylated CpGs.

c ChIP analysis for CTCF binding in WT hESCs, DM1 (SZ-DM14), and edited clones (CL9 and CL29). *FXN* and *APRT* serve as positive and negative controls, respectively. The data is derived from n=3 independent ChIP experiments. Each panel illustrates the average ± standard deviation (STD) calculated across all technical replicates. Statistically significant enrichments were calculated within each cell line for *DMPK* (CTCF1) and *FXN* compared to *APRT* (two-sided paired t-test).

d DNA Sanger sequencing of PCR products covering the breakpoint, post-CTCF ChIP experiment in CL29 cells, shows two alleles in both Input and Bound fractions. Independent allele sequencing after PCR product cloning into a TA-vector served as a control.

e Quantification of total mRNA expression levels for *DMPK* and *SIX5* in WT, DM1-affected, and Δ/Δ hESCs by RT-ddPCR relative to *GUS* expression levels. Each panel averages for *DMPK*, n=7 (CL9 and wild type) or n=8 (DM1 and CL29) and for *SIX5* n=4 (CL29), n=6 (CL9 and wild type) or n=9 (DM1) biological experiments. Error bars represent standard deviation. Statistical significance between cell lines was calculated by pairwise comparison to DM1 affected hESCs (SZ-DM14) (two-sided paired t-test). P-values: ns = $p > 0.05$ *$p < 0.05$, **$p < 0.01$, ***$p < 0.001$, ****$p < 0.0001$). Precise P-values are provided in Table S5.

Source data are provided as a Source data file.

Figure S3

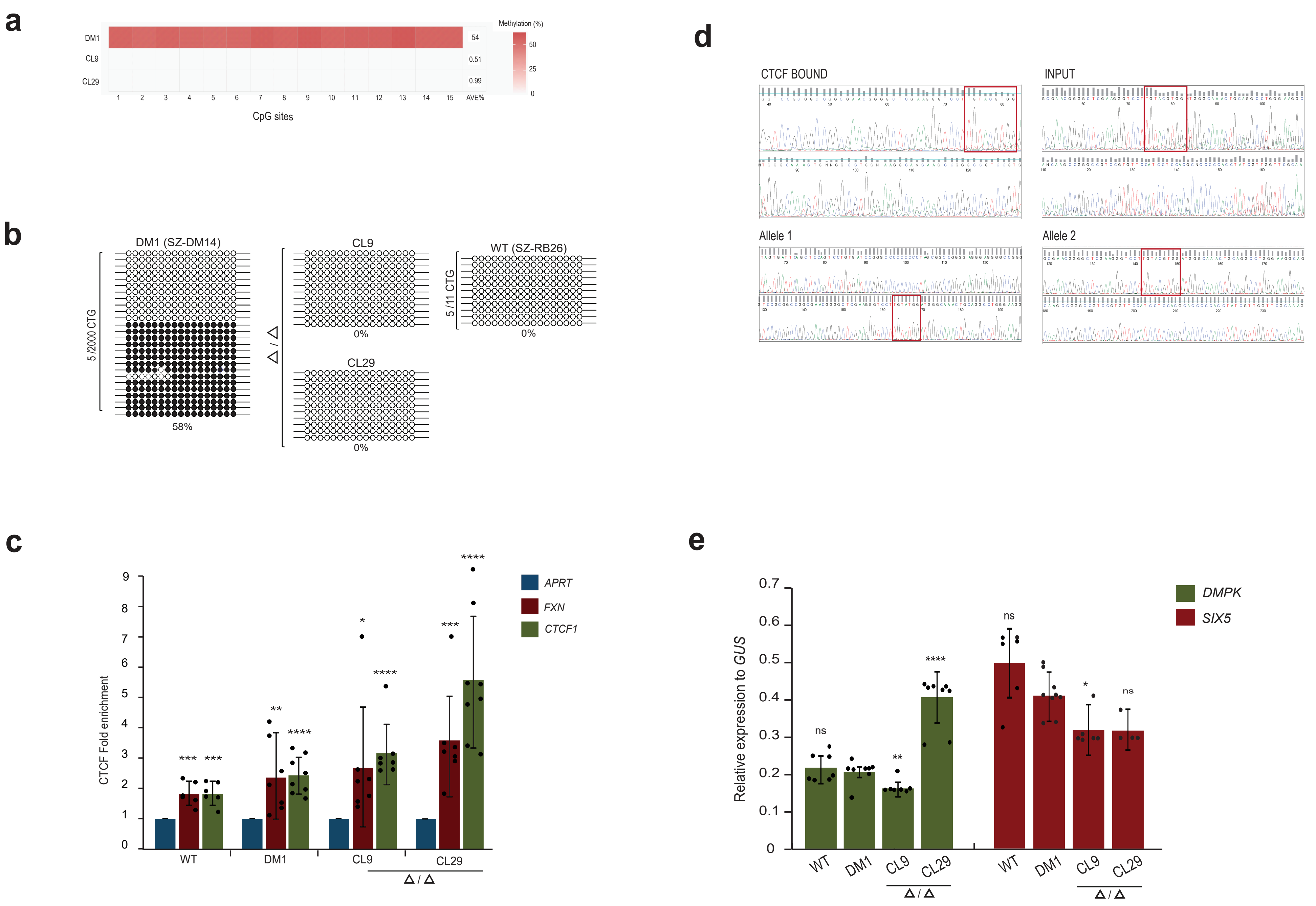

a
DM1
CL9
CL29
54
0.51
0.99
Methylation (%)
50
25
0
1 2 3 4 5 6 7 8 9 10 11 12 13 14 15 AVE%
CpG sites
b
DM1 (SZ-DM14)
5 /2000 CTG
58%
CL9
0%
CL29
0%
Δ / Δ
WT (SZ-RB26)
5 /11 CTG
0%
c
CTCF Fold enrichment
APRT
FXN
CTCF1
WT
DM1
CL9
CL29
Δ / Δ
d
CTCF BOUND
INPUT
Allele 1
Allele 2
e
Relative expression to GUS
DMPK
SIX5
ns
**
****
*
WT
DM1
CL9
CL29
Δ / Δ

Figure S4 - Monitoring for hypermethylation levels after 5-Aza-dC treatment in gene-edited myoblasts. Monitoring for hypermethylation levels by bisulfite sequencing in region E after 5-Aza-dC treatment in gene-edited myoblasts against the background of the mutant allele (variant T) in one unsuccessfully edited (M1) and 4 different gene-edited clones (M4-M7). The left and right panels depict the outcomes of bisulfite DNA colony sequencing before and after a 3-day drug treatment (5 µM). Filled circles: methylated CpGs; empty circles: unmethylated CpGs.

Figure S4

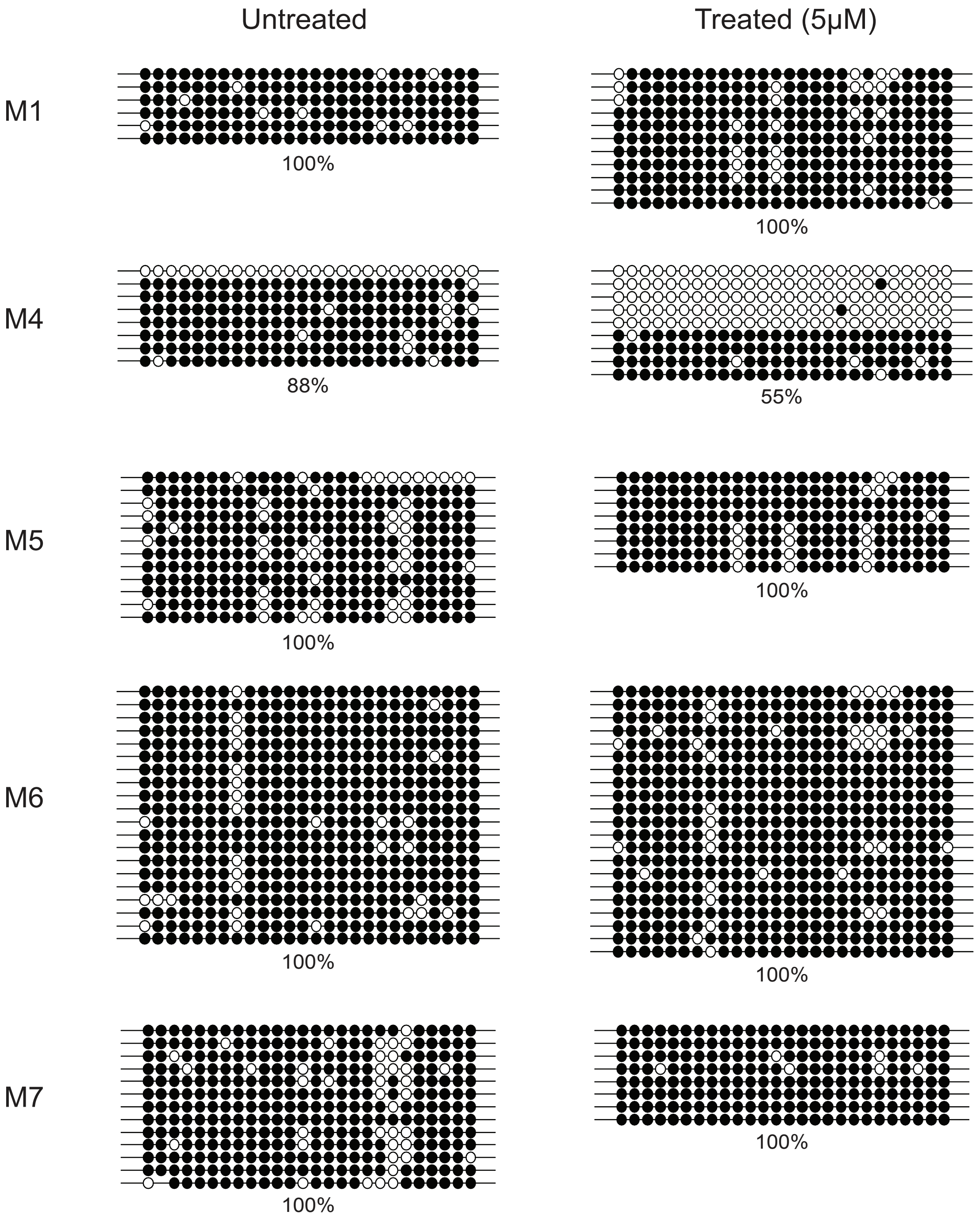
Untreated
Treated (5μM)
M1
100%
100%
M4
88%
55%
M5
100%
100%
M6
100%
100%
M7
100%
100%

Figure S5 - Deletion of the CTGs in fibroblast-like cell cultures derived from DM1 teratomas (CTG2000).

a Validation of successful targeting of the CTGs in the DM1-affected teratoma-derived cell cultures by PCR with a pair of primers that spanned the repetitive region (resulting in a 262 bp product from intact 5CTG allele or a 189 bp product if targeting was successful) demonstrates complete removal of the CTGs in practically 100% of the cells.

b Gene Scan analysis of the PCR products further verified the successful targeting, resulting in a 189 bp fragment due to a 72 bp deletion from the normal (represented by a 262 bp product) or expanded (undetected by PCR) alleles.

c DNA sequencing to confirming accurate repair after gene editing; DNA sequencing at the breakpoint in SZ-DM14 teratoma-derived fibroblast-like cells (CTG2000) before (SZ-DM14 teratoma) and after (DM1 CRISPR) gene editing. The black arrowhead indicates the site where the double strand breaks were fused.

Figure S5

a

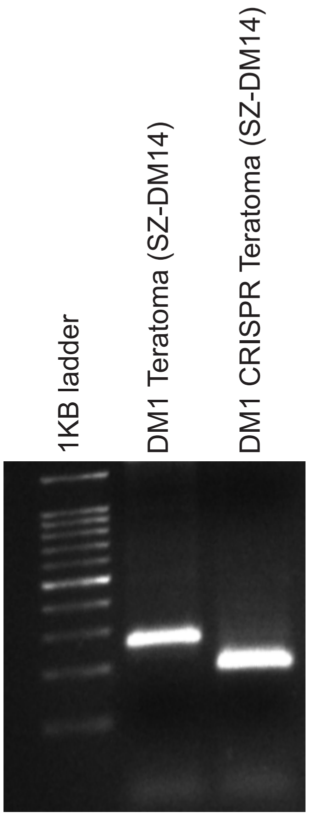

b

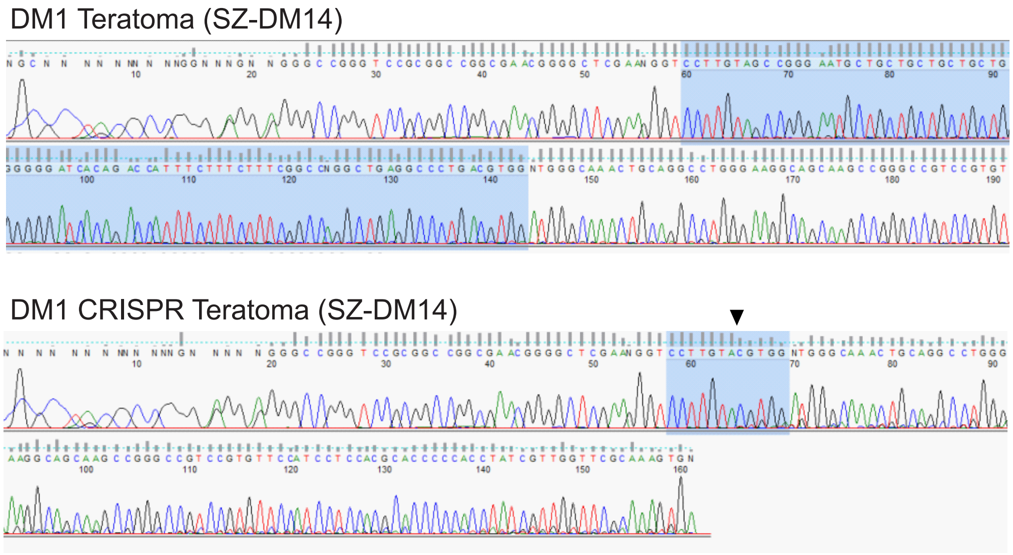

c

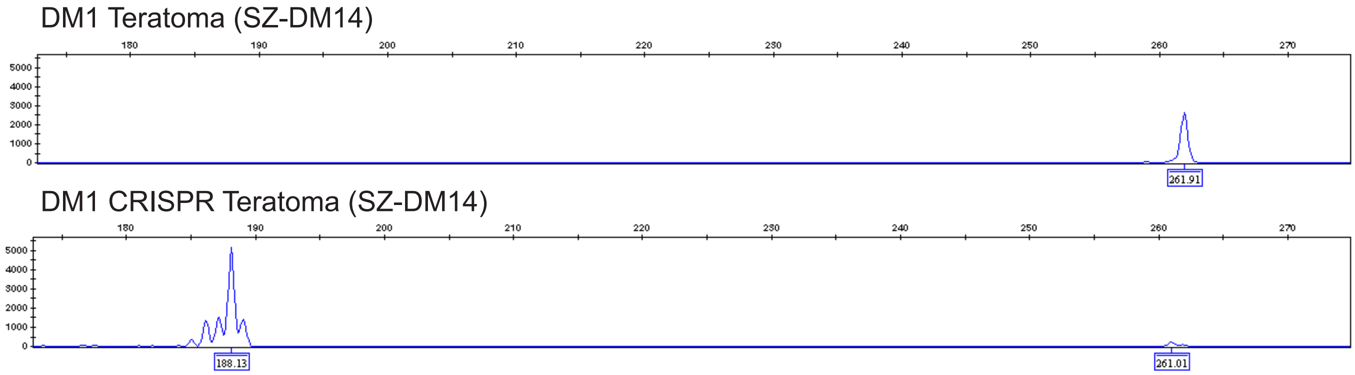

Figure S6 - Derivation of iPSCs from affected gene-edited myoblasts.

a Expression of undifferentiated cell-specific markers by RT-PCR: *OCT4*, *NANOG* and *SOX2* and the housekeeping gene *GAPDH* in iPSCs derived from reprogrammed, repeat-targeted (clones M4 and M6) and untargeted (clone M1) DM1 myoblasts (Table S3).

b Allele-specific colony DNA bisulfite sequencing results in region E after repeat excision in the iPSC clones with (13/2600CTG, clone M1-IPSC1) and without the CTG repeat (Δ/Δ clones M4-IPSC2/3 and M6-IPSC4/5/6). Taking advantage of a non-CpG informative SNP within the DMR served to distinguish between molecules obtained from the normal (variant G) *vs.* the expanded (variant T) alleles in each clone. Methylation patterns against the background of variant T are presented for each iPSC clone. Note that reprogramming did not affect the methylation levels in the region unless the CTG expansion was removed. Filled circles: methylated CpGs; empty circles: unmethylated CpGs.

Figure S6

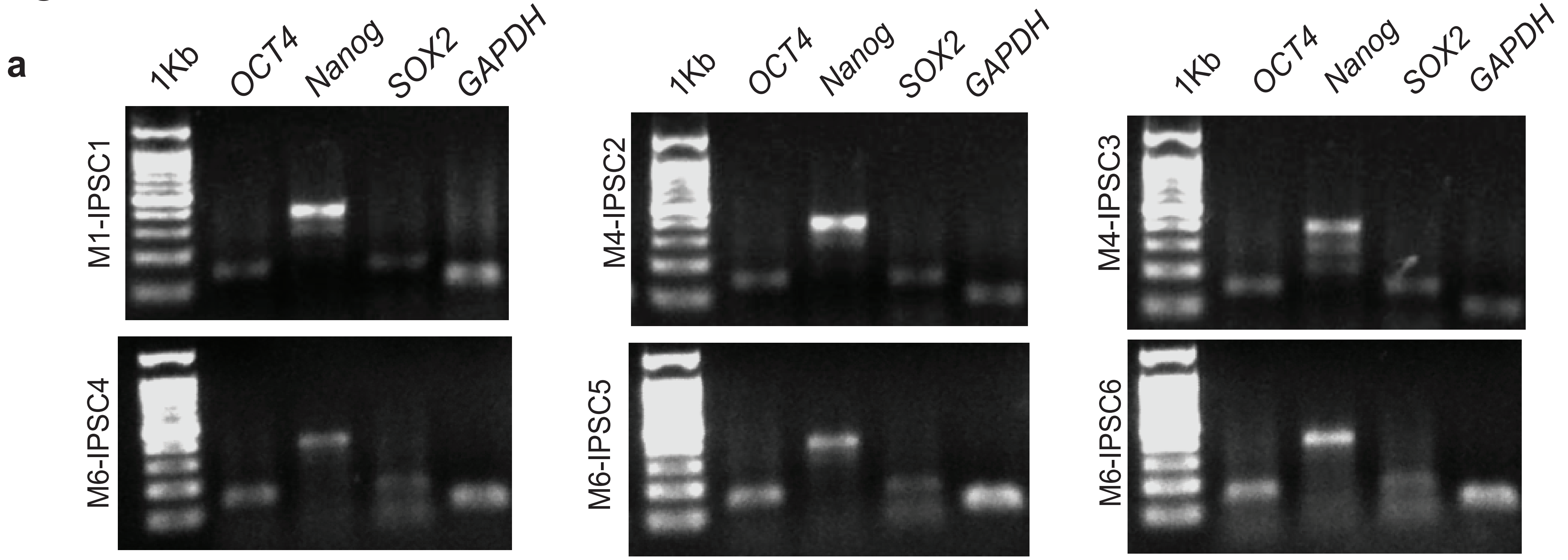
a
1Kb
OCT4
Nanog
SOX2
GAPDH
M1-IPSC1
M4-IPSC2
M4-IPSC3
M6-IPSC4
M6-IPSC5
M6-IPSC6

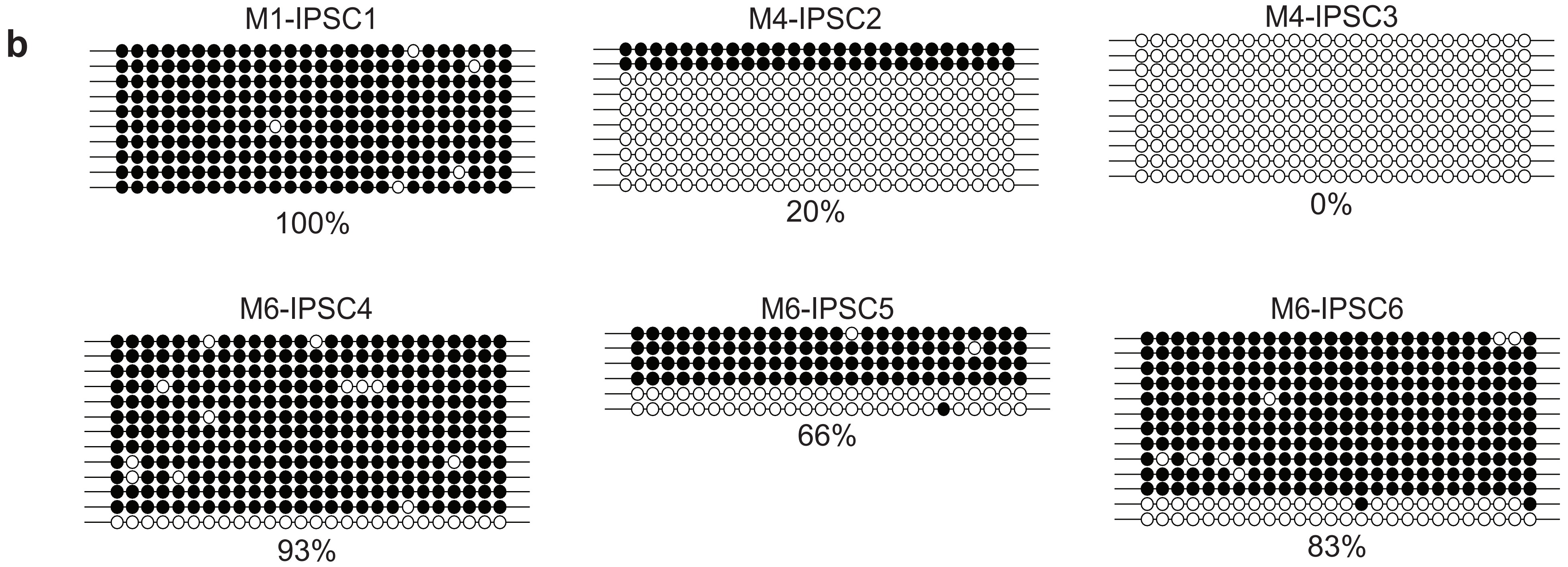
b
M1-IPSC1
100%
M4-IPSC2
20%
M4-IPSC3
0%
M6-IPSC4
93%
M6-IPSC5
66%
M6-IPSC6
83%

Figure S7 - Abnormal methylation at the DM1 locus is maintained by *de novo* DNMTs activity in hESCs

a PCR screening for *DNMT3b* bi-allele deletions in DM1 hESCs. The PCR product size is 401 bp before gene editing, which reduces to 222 bp upon deletion.

b DNA Sanger sequencing across the targeted region in *DNMT3b* confirms the successful induction of the desired deletion and accurate repair. The sites of breakpoint induction is indicated by a red arrow.

c PCR screening for *DNMT3a* bi-allele deletions in *DNMT3b* KO DM1 hESCs. The PCR product size is 497 bp before gene editing, which reduces to 369 bp upon deletion.

d DNA Sanger sequencing across the targeted region in *DNMT3a* confirms the successful induction of the desired deletion and accurate repair. The sites of breakpoint induction is indicated by a red arrow.

e *Top*: cDNA sequence of the catalytic domain of DNMT3a (164 amino acids according to Khrabrova et al. 2019 (35)) covers by a 492 bp fragment that spans over exon 16-20. *Bottom*: cDNA Sanger sequencing following the induction of a bi-allelic deletion in *DNMT3a*, on the background of *DNMT3b*-null DM1 hESC clones, reveals exon 19 skipping in all residual *DNMT3a* mRNA transcripts after gene editing, confirming the remaining of catalytically inactive form of the enzyme. The point of exon skipping is indicated by a red arrow.

Figure S7

a

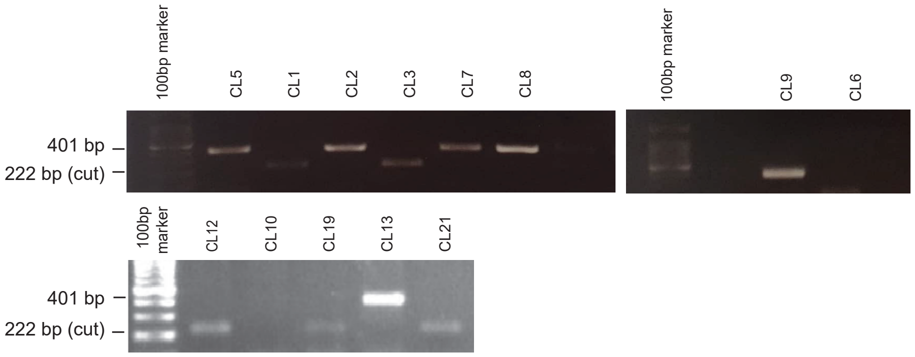

b

DNMT3b gDNA Sanger sequencing

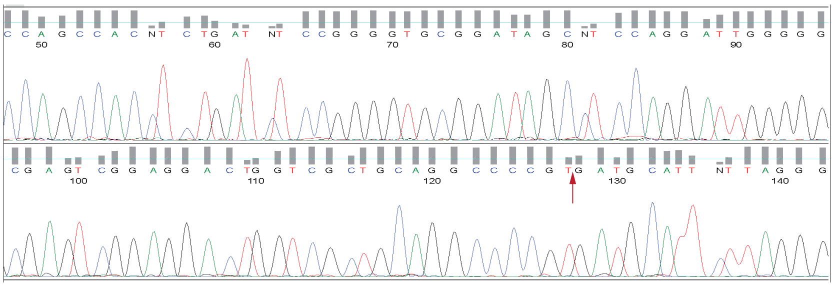

c

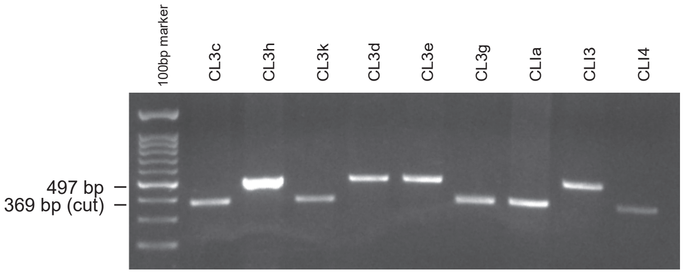

d

DNMT3a gDNA Sanger sequencing

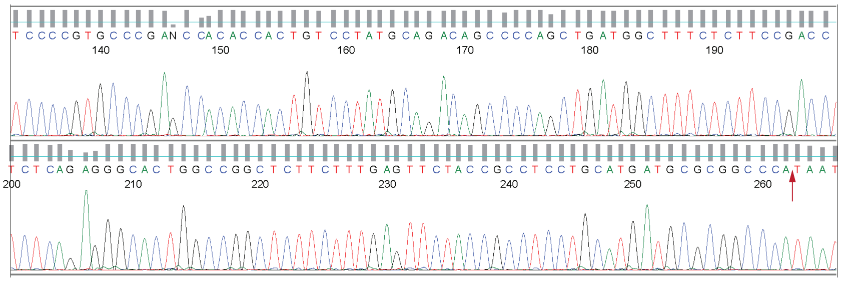

e

mRNA sequence of the catalytic domain of *DNMT3a* (exons 16-20)

ATCCGGGTGCTGTCTCTCTTTGATGGAATCGCTACA**GG**GCTCCTGGTGCTGAAG
GACTTGGGCATTCAGGTGGACCGCTACATTGCCTCGGAGGTGTGTGAGGACTC
CATCACGGTGGGCATGGTGCGGCACCAGGGGAAGATCATGTACGTCGGGGACG
TCCGCAGCGTCACACAGAAGCA**TA**TCCAGGAGTGGGGCCCATTCGATCTGGTG
ATTGGGGGCAGTCCCTGCAATGACCTCTCCATCGTCAACCCTGCTCGCAAGGG
CCTCTAC**GA**GGGCACTGGCCGGCTCTTCTTTGAGTTCTACCGCCTCCTGCATGA
TGCGCGGCCCAAGGAGGGAGATGATCGCCCCTTCTTCTGGCTCTTTGAGAATGT
GGTGGCCATGGGCGTTAGTGACAAGAGGGACATCTCGCGATTTCTCGA**GT**CCAA
CCCTGTGATGATTGATGCCAAAGAAGTGTCAGCTGCACACAGGGCCCGCTACTT
CTGGGGTAAC

Validation of exon 19 skipping by *DNMT3a* cDNA Sanger sequencing

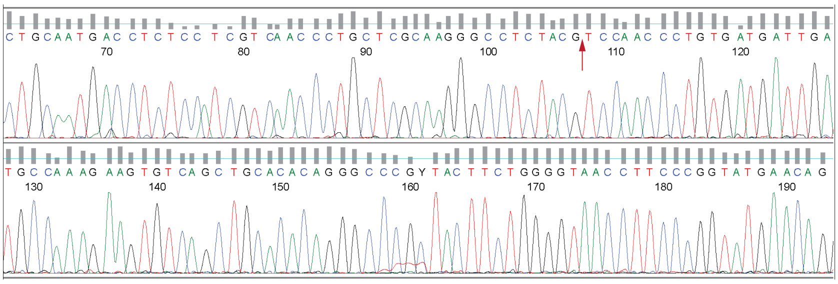

exon 18 exon 20

TCTCCATCGTCAACCCTGCTCGCAAGGGCCTCTAC**GT**CCAACCCTGTGATGATT
GATGCCAAAGAAGTGTCAGCTGCACACAGGGCCCGCTACTTCTGGGGTAAC

Uncropped Southern blots relating to Figure S2a

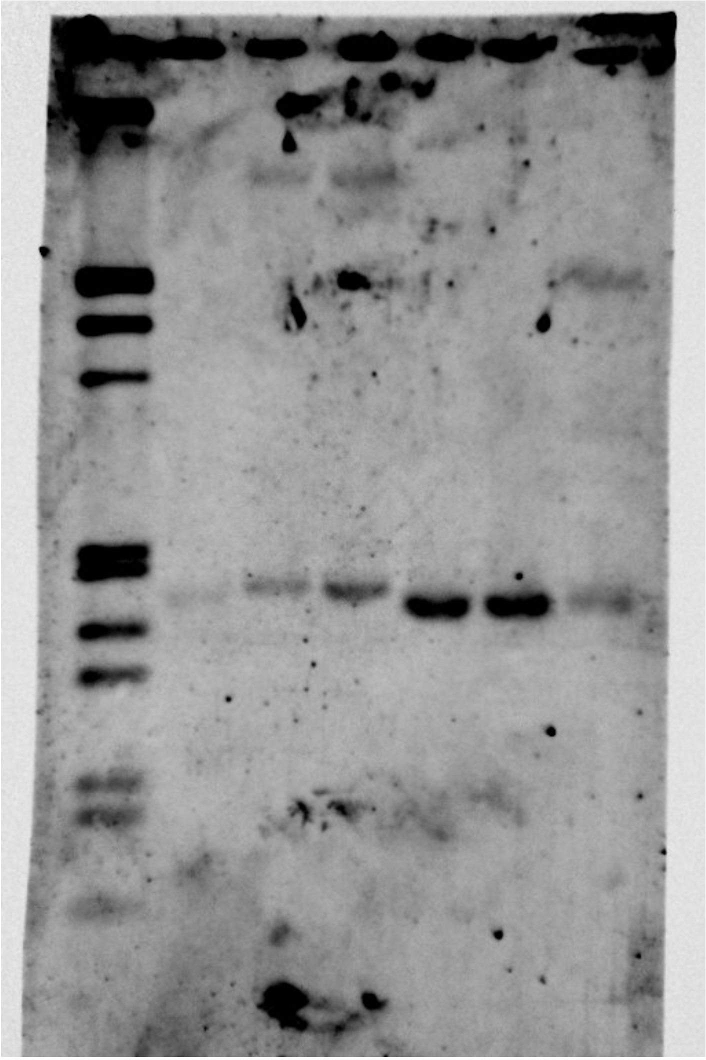

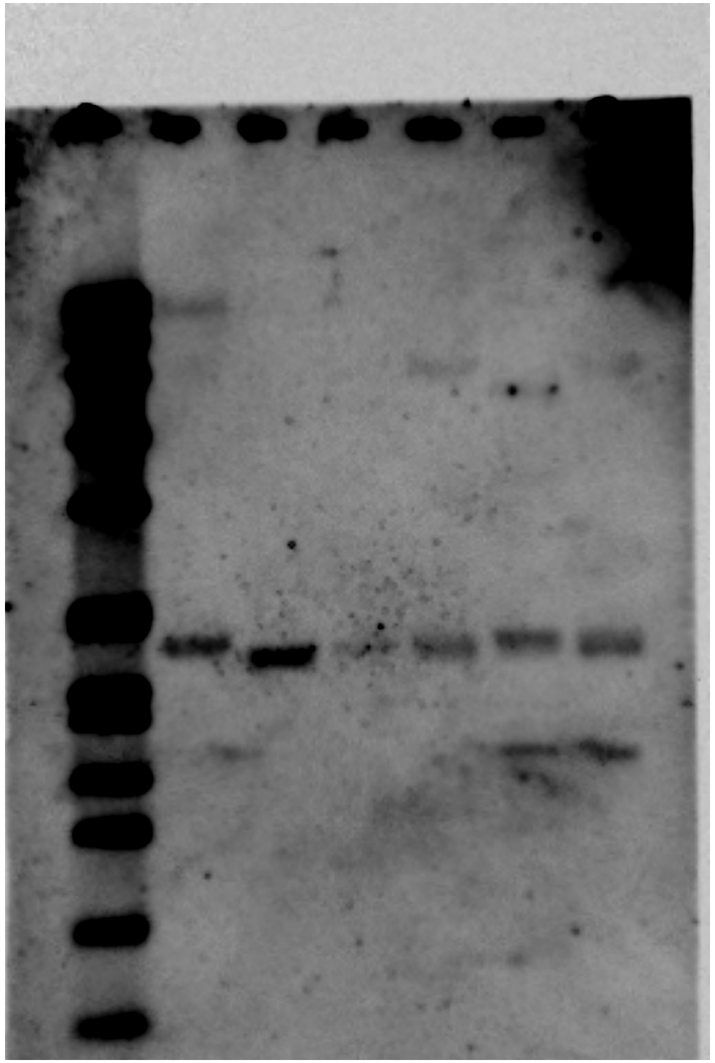

TABLE LEGENDS

Table S1 - Validation of repeat deletion in DM1-affected hESC clones following gene editing by Southern blot analysis and/or Gene Scan analysis of PCR products

Table S2 - gRNA and primer sequences used in this study

Table S3 - List of annotation for DM1-affected myoblast and iPSC clones used in this study

Table S4 - Precise P-values for all experiments

Table S1

Category	Clone #	PCR products	Southern Blot Analysis (CTGs)	Indels
**CTG +/+**	Unmanipulated	262	5/2000	
**CTG Δ/Δ**	9	189/189	Δ/Δ	-
	29	189/189	Δ/Δ	-
**CTG +/Δ**	7	189/262	Δ/5/~1300	-
	10	183/189/262	Δ/5/~1400	+
	12	189/262	5/2000	-
	13	189/262	Δ/5/~700	-
	19	189/262	5/2000	-
	22	189/262	5/2000	-
	25	175/189/262	Δ/5/2000	+
	26	189/262	Δ/5/~1400	-
	11	189/262	ND	-
	14	189/262	ND	-
	15	189/262	ND	-
	17	189/262	ND	-
	20	205/259	ND	+
	27	189/262	ND	-
	28	189/262	ND	-
	31	189/251/262	ND	+

Table S2

Assay	Name primer	5' primer (sequence 5'-3')	3' primer (sequence 5'-3')	Annealing Temp °C	Product size (bp)
**gRNAs**	7gRNA	CAGCAGCATTCCCGGCTACAAGG			
	44gRNA	CAGTTTGCCCATCCACGTCAGGG			
	DNMT3b UP1	CACCGCACCCTAAGAATGCATCCTG			
	DNMT3b Down1	CACCGAGACTCGATCCTCGTCAACG			
	DNMT3a guide 2	CACCGCATGATGCGCGGCCCAAGG			
	DNMT3a guide 4	CACCGTGGTTTGGCCAGCTCACTAA			
**CRISPR validation**	Repeat size	CAGCTCCAGTCCTGTGATCC	CACTTTGCGAACCAACGATA	60	262
	Cut region	GCTAGGAAGCAGCCAATGAC	CATTCCCGGCTACAAGGAC	60	576
	DNMT3b-CRISPR-F/R2new	GAACCAAGTCCTGAGCCTCC	TCGAGCCCTTCACACCTG	60	401
	DNMT3a_long_F/R	TGCAATGACCTCTCCATCGT	GCAAAGCAGAAGTCACCAGT	58	497
**Bisulfite**	E region	TGGTTGTGGGTTAGTGTT	CCCAACAACCTACAACTATTAT	58	289
	F region	AATAATAGTTGTAGGTTGTT	TGTATTGGGTTGGTGGTTTA	56	235
**ChIP qPCR**	*DMPK*	TGCTCCCTGCCAGGGTACG	CCGCAAATGCGCAGCTAAG	60	100
	APRT	GCCTTGACTCGCACTTTTGT	TAGGCGCCATCGATTTTAAG	60	85
	HOXA9	CTCAGGAGCCTCGTGTCT	GTGACCAGGTGGAGGTGT	60	82
	MYOGENIN	GAATCACATCTAATCCACTGTA	ACGCCAACTGCTGGGTGCCA	60	139
	CTCF1	CTGCCAGTTCACAACCGCTCCGAG	GCAGCATTCCCGGCTACAAGGACCCTT	60	147
	FXN	TCCTGAGGTCTAACCTCTAGCTGC	CGAGAGTCCACATGCTGCTCC	60	131
**Off-targets**	*PLEKHJ1*	ACATAGCGAGACCCCATCAC	GGGACCTGGGACTAGACCAT	60	585
	ZFPM1	GTTAATCGCAGCCCTTATCG	TCTGGTTCCTGTCCTTCCAG	60	584
	MTMR2	GCGTAGCCTTCAGAAACCAG	TCCTTATCGCCTTCCTGAGA	60	598
	PIRT	GCTAAGGGAGCTAGGGCTGT	GGACTCATGATGCTGGTGTG	60	617
	SERPINA4	CCAAACCAGGACACCAGAGT	CAAGGCCCTGTAGAGGTCAA	60	606
	EGFR	TCAGAGGGACAGGAAAGGTG	ATGATTCACAAAGGCGGAAG	60	624
	TTC38	TGCTGAGACCTGTTCAGTGC	TGACACATGCCACACTGATG	60	643
	ABHD2	TTGGTGAACAAGGCAGAGTG	AACTGGACAGAGGGCAAGAA	60	609
**RT-PCR**	*OCT4*	GACAGGGGGAGGGGAGGAGCTAGG	CTTCCCTCCAACCAGTTGCCCCAAAC	60	144
	NANOG	CAGCCCCGATTCTTCCACCAGTCCC	CGGAGATTCCCAGTCGGGTTCACC	55	342 and 390
	SOX2	GGGAAATGGGAGGGGTGCAAAAGAGG	TTGCGTGAGTGTGGATGGGATTGGTG	55	151
	GAPDH	ACCCTGTTGCTGTAGCCA	CCACTCCTCCACCTTTGAC	62	102
	exon 19 skipping	AGATCATGTACGTCGGGGAC	GGAAACCAAATACCCTTTCCA	60	573
**RT-qPCR**	*DNMT3a*-RT-Up-ex17/ex18	AGATCATGTACGTCGGGGAC	ATGGAGAGGTCATTGCAGGG	60	110
	GUS	CTCATTTGGAATTTTGCCGATT	CCGAGTGAAGATCCCCTTTTTA	60	81

Table S3

Name	Original Name	Repeat number
**M1**	4F9	13/2600
**M2**	EA11	13/2600
**M3**	EA7	13/2600
**M4**	4A3	No repeats
**M5**	3E3	No repeats
**M6**	3B11	No repeats
**M7**	1E6	13/Δ
**M1-IPSC1**	4F9-2A	13/2600
**M4-IPSC2**	4A3-1A	No repeats
**M4-IPSC3**	4A3-1B	No repeats
**M6-IPSC4**	3B11-3A	No repeats
**M6-IPSC5**	3B11-3B	No repeats
**M6-IPSC6**	3B11-3C	No repeats

Table S4

	ChIP assay			p-value		p-value		p-value		
Figure	Cell type	Clone	Assay	*FXN:APRT*		*DMPK:APRT*				n
				HoxA9:APRT		DMPK:APRT				
Figure1 b	hESCs	wt	H3K9me3	5.32345E-05	****	0.029091911	*			4
		DM1		1.00037E-10	****	1.98633E-07	****			3
		CL9		2.96432E-05	****	0.663799271	ns			3
		CL29		1.614E-07	****	0.001730029	**			3
		Comparisons between cell samples (*DMPK*)								
		WT vs. DM1						2.26E-08	****	
		WT vs. CL9						0.130818917	ns	
		DM1 vs. CL9						5.27E-03	****	
		WT vs. CL29						0.096393802	ns	
		DM1 vs. CL29						1.24618E-06	****	
Figure 1c				*APRT:HOX9*		*APRT:DMPK*				
	hESCs	wt	H3K27me3	2.16736E-06	****	0.032438951	*			3
		DM1		5.06887E-07	****	0.009855714	**			3
		CL9		5.79641E-05	****	0.448734061	ns			3
		CL29		0.000456586	***	0.191048422	ns			3
		Comparisons between cell samples (*DMPK*)								
		WT. vs. DM1						0.919799201	ns	
		CL9 vs. DM1						0.470681444	ns	
		CL29 vs. DM1						0.908557351	ns	
		CL9 vs. WT						0.621730936	ns	
		CL29 vs. WT						0.987902001	ns	
Figure 2b				*MYOG:APRT*		*DMPK:APRT*				
	Myoblasts	M6	H3K9me3	1.92572E-07	****	2.05532E-05	****			4
		M4		5.33088E-05	****	0.001177222	**			3
		M1		5.88196E-09	****	0.000619777	***			4
		M3		4.55504E-06	****	4.74065E-06	****			4
Figure 2c				*DMPK:APRT*						
	Myoblasts	M6	H3K27me3	0.000139816	***					2
		M4		6.6624E-07	****					3
		M1		3.2151E-08	****					5
		M3		9.24496E-08	****					3
Figure S3c	hESCs	wt	CTCF	0.000619054	***	0.000452637	***			3
		DM1		0.008959111	**	2.08809E-05	****			3
		CL9		0.044454734	*	0.00010102	****			3
		CL29		0.00071118	***	3.62197E-05	****			3

Gene Expression

RT- droplet PCR

	cell type	clone	gene		p-value		n
			DMPK				
Figure S3e	hESCs	wt					7
		DM1		DM1:WT	0.554713148	ns	8
		CL9		DM1:cl9	0.008350344	**	7
		CL29		DM1:cl29	6.4374E-06	****	8
			SIX5				
		wt					6
		DM1		DM1:WT	0.051913073	ns	9
		CL9		DM1:cl9	0.044687155	*	6
		CL29		DM1:cl29	0.066847117	ns	4

RT-qPCR

	cell type	clone	gene	p-value		n
Figure 4d			*Dnmt3a*	clone:ctl		
	hESCs	CTRL (DM1)				6
		Cl-3c		3.51242E-08	****	6
		Cl-3k		0.000225917	***	6
		Cl-3g		2.0407E-07	****	5
		Cl-I3		3.34153E-07	****	4
		Cl-I4		1.57131E-06	****	6
		Myob(M1)		2.50152E-07	****	3
