# Supplementary material for: Differentiation shifts from a reversible to an irreversible heterochromatin state at the DM1 locus
Source: Nat Commun. 2024 Apr 16;15:3270. doi: 10.1038/s41467-024-47217-4 (PMC11021500; doi:10.1038/s41467-024-47217-4)
Supplement: Supplementary file 3 — Description of Additional Supplementary Files [file 41467_2024_47217_MOESM3_ESM.pdf]

Description of Additional Supplementary Files

File Name: Supplementary Data 1
Description: List of all chromatin modifiers with corresponding expression levels.
